# Effect of ALDH2 on High Glucose-Induced Cardiac Fibroblast Oxidative Stress, Apoptosis, and Fibrosis

**DOI:** 10.1155/2017/9257967

**Published:** 2017-10-09

**Authors:** Xiaoyu Gu, Tingting Fang, Pinfang Kang, Junfeng Hu, Ying Yu, Zhenghong Li, Xiangyang Cheng, Qin Gao

**Affiliations:** ^1^Department of Physiology, Bengbu Medical College, Bengbu Anhui 233030, China; ^2^Science Research Centre, Bengbu Medical College, Bengbu, Anhui 233030, China; ^3^Department of Anesthesiology, The First Affiliated Hospital of Bengbu Medical College, Bengbu Anhui 233004, China; ^4^Department of Cardiovascular Disease, The First Affiliated Hospital of Bengbu Medical College, Bengbu Anhui 233004, China; ^5^Department of Respiratory, The First Affiliated Hospital of Bengbu Medical College, Bengbu, Anhui 233004, China

## Abstract

Our study aimed firstly to observe whether ALDH2 was expressed in neonate rat cardiac fibroblasts, then to investigate the effect of activation of ALDH2 on oxidative stress, apoptosis, and fibrosis when cardiac fibroblasts were subjected to high glucose intervention. Cultured cardiac fibroblasts were randomly divided into normal (NG), NG + Alda-1, high glucose (HG), HG + Alda-1, HG + Alda-1 + daidzin, HG + daidzin, and hypertonic groups. Double-label immunofluorescence staining, RT-PCR, and Western blot revealed ALDH2 was expressed in cardiac fibroblasts. Compared with NG, ALDH2 activity and protein expression were reduced, and cardiac fibroblast proliferation, ROS releasing, 4-HNE protein expression, collagen type I and III at mRNA levels, and the apoptosis rate were increased in HG group. While in HG + Alda-1 group, with the increases of ALDH2 activity and protein expression, the cardiac fibroblast proliferation and ROS releasing were decreased, and 4-HNE protein expression, collagen type I and III at mRNA levels, and apoptosis rate were reduced compared with HG group. When treated with daidzin in HG + Alda-1 group, the protective effects were inhibited. Our findings suggested that ALDH2 is expressed in neonate rat cardiac fibroblasts; activation of ALDH2 decreases the HG-induced apoptosis and fibrosis through inhibition of oxidative stress.

## 1. Introduction

Diabetes mellitus (DM) is one of the most serious chronic diseases in the world. The International Diabetes Federation (IDF) estimated [1] that DM will be one of the most serious causes of death by 2030, and the number of adults who suffered from DM will rise highly to 642 million by 2040. Diabetic cardiomyopathy (DCM) is one of the main complications of DM, which contributes to the high fatality rate in DCM patients. Myocardial fibrosis is a popular pathological process of DCM.

In previous studies, scientists usually focused on cardiomyocyte in myocardial injury *in vivo* [2, 3]. However, it is a widely recognized myocardial injury, especially myocardial fibrosis, which is closely related to the pathophysiological changes of cardiac fibroblasts (CFs) [4]. Myocardial pathological remodeling involves not only the reactivation of cardiomyocyte death but also CF proliferation and ECM expression. Cardiac fibroblasts are the main components of the heart besides cardiomyocytes, vascular endothelial cells, and vascular smooth muscle cells [5]. CFs have the strong proliferation capacity, and the number is about two times of the cardiomyocytes in the heart [6]. Cardiac fibroblasts are the primary cell type responsible for synthesis, deposition, and degradation of extracellular matrix (ECM) proteins. ECM is no longer considered a static support structure for cells, but a dynamic signaling network with the power to influence cells, tissues, and whole organ physiology. Therefore, ECM proteins play a critical role in the development and maintenance of functional heart tissue, and the changes of cardiac fibroblast function will lead to heart failure. Among the main components of ECM, collagens play the important role in myocardium remodeling. Cardiac fibrosis is a final common pathway in many cardiovascular diseases, which is characterized by the proliferation of CFs and excessive deposition of ECM [7]. Therefore, it is critical to investigate the role and mechanism of CFs on myocardial fibrosis.

Acetaldehyde dehydrogenase 2 (ALDH2) is a member of 19 ALDH gene families. It plays a crucial role in the inhibition of oxidative stress and the detoxification of reactive aldehydes such as 4-hydroxy-2-nonenal (4-HNE) [8]. Our previous studies had reported that increasing ALDH2 expression can ameliorate myocardial ischemia/reperfusion injury and diabetes mellitus-induced myocardial injury [9–11]. Other papers also showed that ALDH2 can attenuate cardiac injury induced by toxic metabolites [12], and activation of ALDH2 inhibited AMPK activation, increased the phosphorylation of FOXO3a (Forkhead box O3), and reduced myocardial apoptosis by high glucose-induced myocardial injury [13]. The activation of ALDH2 can resist the excessive production of oxygen radicals caused by various kinds of myocardial injury and apoptosis; however, these studies mainly focused on cardiomyocyte injury and paid few attentions on cardiac fibroblasts. Our previous results showed that myocardial fibrosis happened in a rat DM model with the increases of hydroxyproline, the collagen deposition, and the failure of myocardial systolic and diastolic dysfunction [10]. So, is ALDH2 expressed in myocardial fibroblast? If it is expressed, was ALDH2 downregulated in high glucose-induced myocardial fibroblast injury? And can increasing ALDH2 expression protect myocardial fibroblast against high glucose-induced injury? The underlying mechanisms are still not clearly understood.

So, based on our previous study [9–11], we offered the hypothesis in this study: ALDH2 is expressed in neonate rat cardiac fibroblasts; then, activation of ALDH2 can attenuate high glucose-induced myocardial fibroblast injury. We selected 30 mM glucose to induce neonate rat cardiac fibroblast injury, to observe the expression of ALDH2, and to investigate the likely mechanisms of ALDH2 on cardiac fibroblast injury.

## 2. Materials and Methods

### 2.1. Isolation, Primary Culture, and Identification of Cardiac Fibroblasts

The apexes of the hearts were isolated from 1 ~ 3-day-old Sprague-Dawley rats which were obtained from the Experimental Animal Center of Bengbu Medical College (Bengbu, China). All the animal procedures were in accordance with the United States National Institutes of Health Guide and were approved by the Animal Use and Care Committee of Bengbu Medical College.

After washing in precooling D-Hank's solution, the heart tissues were sheared and fully digested (37°C, 5% CO_2_, incubated for 7 ~ 8 min). The digestive enzymes consisted of trypsin (0.07%, Beyotime Biotechnology, Shanghai, China), type II collagenase (0.08%, Sigma-Aldrich Co., St. Louis, MO, USA), and DNase I (10 *μ*g/mL, Beijing Solarbio Science & Technology Co. Ltd., Beijing, China). When the tissue started to loosen, precooling DMEM medium (10% FBS) was added to stop the digestion. Cells isolated from the tissue were collected and cultured in DMEM medium (glucose 5.5 mM) containing 10% FBS in an incubator (37°C, 5% CO_2_) for 90 min. Vimentin (1 : 200, Boster Biological Technology, Wuhan, China)-positive cells were considered as CFs. As the cells grew to 80% confluence, they were passaged at a ratio of 1 : 2, and the 2nd to 4th passages of cells were used for the following experiments.

### 2.2. Double-Label Immunofluorescence Staining

ALDH2 expression of CFs was detected by double-label immunofluorescence staining technique. Anti-vimentin antibody (1 : 200, Boster Biological Technology, Wuhan, China) and rabbit anti-ALDH2 antibody (1 : 100, Abcam Co., Cambridge, UK) were used in the experiment operation. Cell slides were incubated with mixed primary antibody overnight at 4°C after blocking with 5% bovine serum albumin (BSA) for 30 min at 37°C. The mixed secondary antibodies were added and incubated in 37°C for the optimized time and dilution. The nuclei of CFs were stained with DAPI for 10 min in 37°C. Fluorescent images were obtained with fluorescence microscope camera (OLYMPUS U-HGLGPS, Japan).

### 2.3. Identification of ALDH2 Expression in CFs

For identifying whether ALDH2 is expressed in CFs, ALDH2 mRNA and protein expressions were detected by reverse transcription polymerase chain reaction (RT-PCR) and Western blot. Total RNA from CFs was isolated using TRIzol reagent (Invitrogen, Grand Island, NY, USA), and 50 ng of total cDNA was used for PCR analysis with PCR Master Mix (2×) (K0171, Thermo Fisher Scientific Inc., New York, USA), after reverse transcription using RevertAid RT Reverse Transcription Kit (Thermo Fisher Scientific Inc., New York, USA). The thermal cycling conditions of RT-PCR are as follows: 95°C for 3 min, then 40 cycles of 95°C for 30 sec, 62.5°C for 30 sec, and 72°C for 35 sec, followed by a final extension step at 72°C for 10 min.

### 2.4. Experimental Grouping

CFs were divided into 7 groups after incubated (37°C, 5% CO_2_) in a serum-free DMEM medium for 48 h:

Group 1: Normal group (NG), CFs were cultured with DMEM medium with normal glucose (glucose concentration at 5.5 mM) and treated with the same volume of solvent instead of drug.
Group 2: Normal glucose group + Alda-1 (NG + Alda-1), Alda-1 at 20 *μ*M (the specific agonist of ALDH2, Sigma-Aldrich Co., St. Louis, MO, USA) [14] was added into DMEM medium with normal glucose and cultured for 48 h.
Group 3: High glucose groups (HG), CFs were cultured in DMEM medium with high glucose (glucose concentration at 30 mM) to induce injury for 48 h.
Group 4: HG + Alda-1 group (HG + Alda-1), for observing whether activation of ALDH2 can attenuate HG-induced CF injury, 20 *μ*M Alda-1 was added into DMEM medium with 30 mM glucose and cultured for 48 h.
Group 5: HG + Alda-1 + daidzin (HG + Alda-1 + daidzin), 20 *μ*M Alda-1 and 60 *μ*M daidzin (the specific antagonist of ALDH2, Sigma-Aldrich Co., St. Louis, MO, USA) [15] were added into DMEM medium with 30 mM glucose and cultured for 48 h.
Group 6: HG + daidzin group (HG + daidzin), 60 *μ*M daidzin was added into DMEM medium with 30 mM glucose and cultured for 48 h.
Group 7: Hypertonic group (HPG), for excluding the role of hypertonic, CFs were cultured with DMEM medium with 5.5 mM glucose and treated with 24.5 mM mannitol 48 h.

### 2.5. MTT Measurement

MTT measurement was done in all groups. CFs were seeded in 96-well plates at a density of 1 × 10^6^ cells/plate. Cell viability was assessed in seven different groups using MTT assay (Biosharp, Hefei, China) according to the manufacturer. The optical density (OD) values of the cells in each well of different groups were measured at 490 nm.

DHE staining, qRT-PCR, Western blot, and apoptosis measurements were done in six groups excluding hypertonic group.

### 2.6. ROS Level Detected by DHE

Superoxide production in the CFs was detected by dihydroethidium (DHE, Sigma-Aldrich Co., St. Louis, MO, USA) staining. CFs were seeded in 6-well plates at a density of 1 × 10^6^ cells/plate. The CFs were incubated with 1 *μ*M DHE solution at 37°C for 30 min away from light. Fluorescent images were obtained with fluorescence microscope camera (OLYMPUS U-HGLGPS, Japan) and the mean fluorescence intensity was analyzed with ImageJ software.

### 2.7. ALDH2 Activity Detection

The ALDH2 activity was assessed using the mitochondrial aldehyde dehydrogenase (ALDH2) activity assay kit (ab115348, Abcam Co., Cambridge, UK). Briefly, the activity is determined by following the production of NADH in the following ALDH2 catalyzed reaction: acetaldehyde + NAD^+^ → acid + NADH, we determined the activity of ALDH2 by measuring absorbance of acid at 450 nm. All reagents were provided and we conducted the experiment according to the manufacturer's protocol.

### 2.8. Reverse Transcription Real-Time PCR (qRT-RCR)

Total RNA from CFs was isolated using TRIzol reagent (Invitrogen, Grand island, NY, USA), and 50 ng of total cDNA was used for real-time PCR analysis with SYBR® Premix DimerEraser™ Kit (Takara Biotechnology (Dalian) Co. Ltd., Dalian, China) after reverse transcription using RevertAid RT Reverse Transcription Kit (Thermo Fisher Scientific Inc., New York, USA). The thermal cycling conditions of real-time PCR are as follows: 95°C for 3 sec, then 40 cycles of 95°C for 5 sec, 59°C for 30 sec, and 72°C for 34 sec. The primers we purchased from Sangon Biotech (Shanghai, China) listed in Table 1. Gene expression was normalized to the endogenous control (GAPDH mRNA), and the amount of target gene mRNA expression in each sample was expressed relative to that of the control. The details of Western blot were explained in the following text.

### 2.9. Western Blot Analysis of ALDH2 and 4-HNE

CFs in each group were collected and homogenized in RIPA lysis buffer (Beyotime Biotechnology, Shanghai, China) (add PMSF 0.1 mM) for 1 hour on ice. The lysates were centrifuged at 10,000 rpm and 4°C for 15 min, and the supernatants were used for Western blot after protein quantification. Proteins were separated by SDS-PAGE and blotted onto polyvinylidene fluoride (PVDF) membranes [16]. After blocking by nonfat milk for 2.5 h, immunoblotting was performed with the following antibodies: anti-GAPDH antibody (36 kDa, 1 : 1000, Boster Biological Technology, Wuhan, China) as a control for loading, rabbit anti-ALDH2 antibody (56 kDa, 1 : 3000, Abcam Co., Cambridge, UK), and 4-HNE (70 kDa, 1 : 2000, Abcam Co., Cambridge, UK). Detection and quantification were performed by ECL with horseradish peroxidase- (HRP-) linked anti-rabbit IgG (1 : 10,000, Boster Biological Technology, Wuhan, China). Densitometric quantification of antibody-specific dots was performed with ChemiDoc™ XRS+ System and analyzed with Tanon software (version 4.2.1).

### 2.10. Flow Cytometry

Apoptosis rate was detected by Annexin V and propidium iodide double staining method through flow cytometry by Annexin V-FITC Apoptosis Detection Kit (Kaiji Biological Engineering Co. Ltd., Nanjing, China). CFs in each group were collected and resuspended in 500 *μ*L binding buffer. CFs were labeled with Annexin V-FITC and propidium iodide (PI) and then incubated for 15 min in the dark. All samples were analyzed by flow cytometry.

### 2.11. Statistical Analyses

All data analyses were performed using SPSS software (version 16.0) and expressed as means ± SEM. One-way ANOVA analysis (Newman-Keuls for comparisons of multiple means) was used for statistical analyses. *P* values <0.05 were considered as statistically significant.

## 3. Results

### 3.1. The Identification of CFs

**Figure 1:**
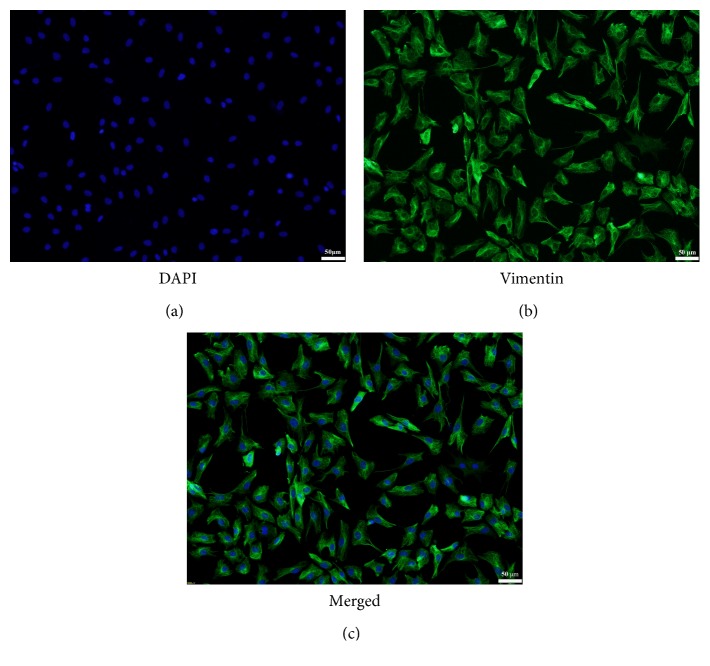
The identification of cardiac fibroblasts by immunofluorescence technique. (a) Nuclei of CFs were stained with DAPI (magnification ×100). (b) The cytoplasm was stained green which showed that CFs can be identified with the positive expression of vimentin (magnification ×100). (c) The merged picture of (a) and (b) (magnification ×100).

Vimentin is a kind of intermediate fiber in interstitial cells, which is an integral part of the cytoskeleton. It maintains the integrity of the cells. It is reported that cardiac fibroblasts can be identified with the positive expression of vimentin. The results showed that vimentin-positive cells with green fluorescence were considered highly purified cardiac fibroblasts (Figure 1).

### 3.2. The Detection of ALDH2 in CFs

**Figure 2:**
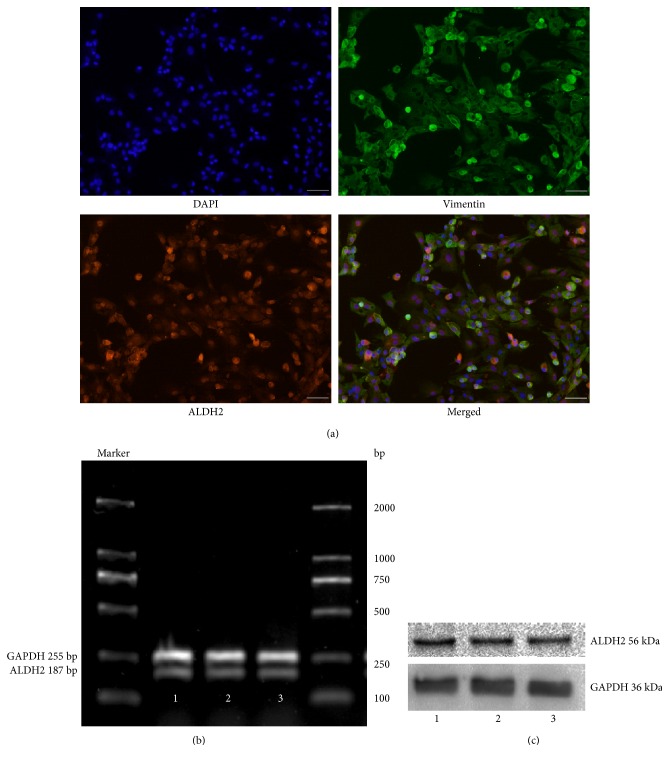
The ALDH2 expression of cardiac fibroblasts detected by double-label immunofluorescence-staining technique (magnification ×100) (a). Red fluorescence-positive cells indicated that ALDH2 was expressed in CFs. The mRNA (b) and protein (c) expressions of ALDH2 were detected by RT-PCR and Western blot in three different batches of CFs. GAPDH was used as a loading control.

For identifying whether ALDH2 is expressed in CFs, double-label immunofluorescence staining technique was used to observe cellular localization of ALDH2. RT-PCR and Western blot methods were used, respectively, to measure ALDH2 expression at mRNA and protein level in three different batches of CFs. ALDH2-positive cells with red fluorescence indicated that ALDH2 was expressed in CFs (Figure 2(a)). Meanwhile, ALDH2 at mRNA (Figure 2(b)) and protein (Figure 2(c)) levels were expressed in cardiac fibroblasts.

### 3.3. MTT Measurement

**Figure 3:**
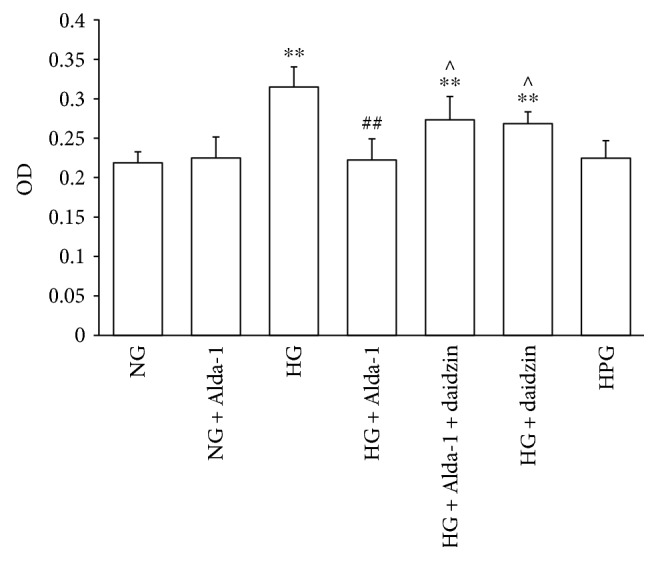
The OD value of CFs in different experimental groups were measured by MTT method. Data were presented as the mean ± SEM (*N* = 6). ^∗∗^*P* < 0.01 versus NG, ^##^*P* < 0.01 versus HG, ^^^*P* < 0.05 versus HG + Alda-1.

There was no significant difference in cell viability and proliferation of CFs among normal group (NG), NG + Alda-1, and HPG groups, so HPG intervention was not used in the mechanism research. Cell viability and proliferation of CFs in HG, HG + Alda-1 + daidzin, and HG + daidzin were higher than those of cells in NG (*P* < 0.01). When compared with HG, the viability and proliferation in HG + Alda-1 were inhibited (*P* < 0.01). The viability and proliferation were increased (*P* < 0.05) in HG + Alda-1 + daidzin and HG + daidzin groups compared with HG + Alda-1 (Figure 3).

### 3.4. The Levels of ROS by DHE Staining

**Figure 4:**
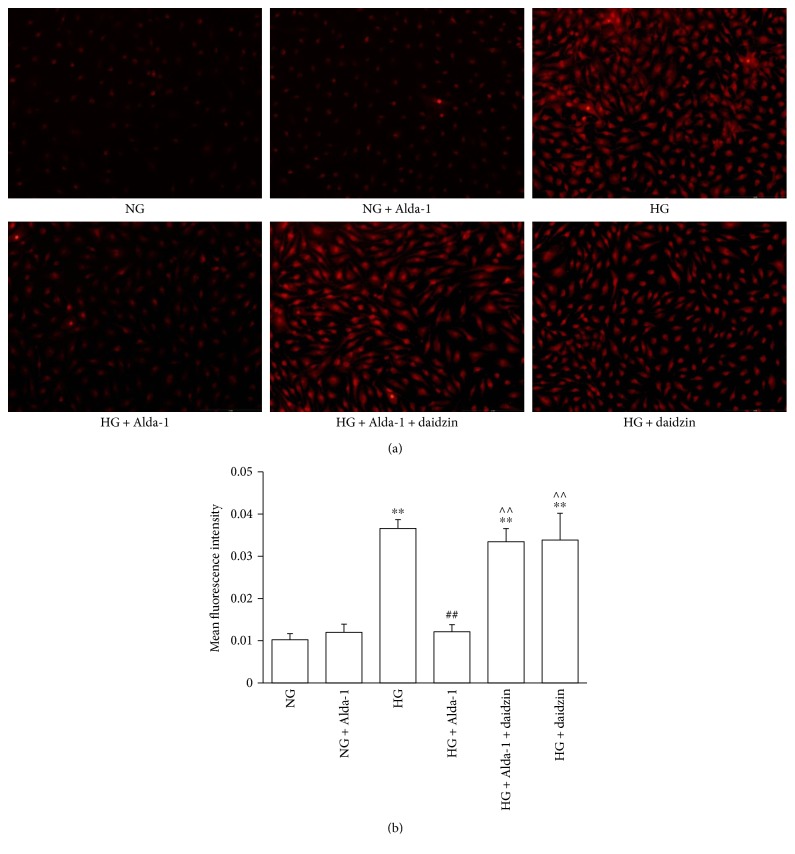
ROS level of CFs in different experimental groups. (a) Intracellular ROS accumulation in different groups stained by DHE. Representative pictures were shown. (b) Mean fluorescence intensity was detected by ImageJ software. Data were presented as the mean ± SEM (*N* = 3). ^∗∗^*P* < 0.01 versus NG, ^##^*P* < 0.01 versus HG, ^^^^*P* < 0.01 versus HG + Alda-1.

Compared with NG group, there was no change of DHE fluorescence intensity in NG + Alda-1 group, but DHE fluorescence intensity was enhanced significantly in HG group. When compared with HG group, DHE fluorescence intensity was obviously weak in HG + Alda-1 group and was stronger in HG + Alda-1 + daidzin and HG + daidzin groups (Figures 4(a) and 4(b)).

### 3.5. Changes of ALDH2 Activity in Each Group

**Figure 5:**
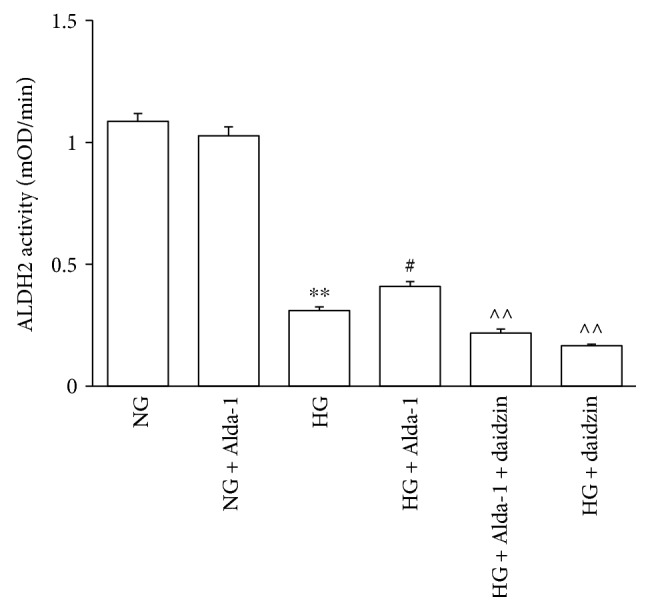
ALDH2 activity in different experimental groups. Data were presented as mean ± SEM (*N* = 6). ^∗∗^*P* < 0.01 versus NG, ^#^*P* < 0.05 versus HG, ^^^^*P* < 0.01 versus HG + Alda-1.

ALDH2 activity was assessed in primary CFs isolated from neonatal rats (Figure 5). The results showed there was no significant difference of ALDH2 activity between NG and NG + Alda-1 groups. Compared with NG group, the activity of ALDH2 was decreased in HG group. When compared with HG group, the activity of ALDH2 was increased in HG + Alda-1 group. The activity of ALDH2 was reduced significantly in HG + Alda-1 + daidzin and HG + daidzin groups compared with HG + Alda-1 group.

### 3.6. Changes of the Expressions of Collagen I and Collagen III at mRNA Level

**Figure 6:**
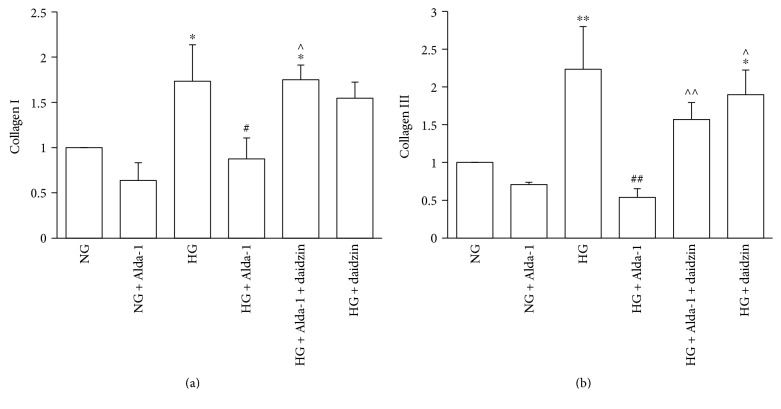
Expressions of collagen I and collagen III at mRNA level in the different experimental groups. mRNA levels of collagen I (a) and collagen III (b) were quantified by real-time-PCR analysis in the CFs of each experimental group. GAPDH was used as a loading control. Data were presented as the mean ± SEM (*N* = 3). ^∗^*P* < 0.05, ^∗∗^*P* < 0.01 versus NG, ^#^*P* < 0.05, ^##^*P* < 0.01 versus HG, ^^^*P* < 0.05, ^^^^*P* < 0.01 versus HG + Alda-1.

There was no significant difference of the expressions of collagen I and collagen III at mRNA level in NG and NG + Alda-1 groups. When compared with NG group, the mRNA expressions of collagen I and collagen III were increased (*P* < 0.01) in HG group. However, collagen I and collagen III mRNA expressions in HG + Alda-1 group were decreased compared with HG group (*P* < 0.01). When compared with HG + Alda-1 group, the mRNA expressions of collagen I and collagen III in HG + Alda-1 + daidzin and HG + daidzin groups were increased (*P* < 0.05) (Figures 6(a) and 6(b)).

### 3.7. Changes of the Expressions of 4-HNE and ALDH2 at Protein Level

**Figure 7:**
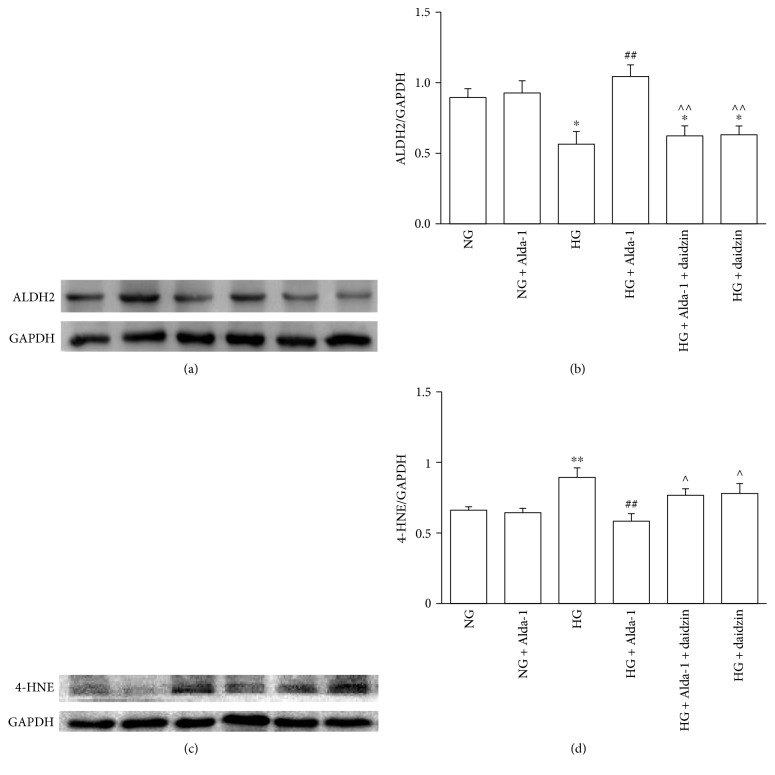
The changes of ALDH2 and 4-HNE protein levels in different experimental groups. (a) Representative blots of ALDH2 and GAPDH in CFs. (b) ALDH2 protein levels in CFs normalized by GAPDH levels and all the data were presented as mean ± SEM (*N* = 8). (c) Representative blots of 4-HNE and GAPDH in CFs. (d) 4-HNE protein levels in CFs normalized by GAPDH levels and all the data were presented as mean ± SEM (*N* = 5). ^∗^*P* < 0.05, ^∗∗^*P* < 0.01 versus NG, ^##^*P* < 0.01 versus HG, ^^^*P* < 0.05, ^^^^*P* < 0.01 versus HG + Alda-1.

There was no significant difference of 4-HNE and ALDH2 in NG and NG + Alda-1 groups. When compared with NG group, ALDH2 protein expression in HG group was decreased (*P* < 0.01), while 4-HNE expression was increased (*P* < 0.01). The protein expression of ALDH2 in HG + Alda-1 group was increased (*P* < 0.01), while the protein expression of 4-HNE was decreased (*P* < 0.01) compared with HG group. When compared with HG + Alda-1 group, the protein expression of ALDH2 were decreased (*P* < 0.01), and the protein expression of 4-HNE was increased (*P* < 0.05) in HG + Alda-1 + daidzin, and HG + daidzin groups were decreased (*P* < 0.01) while the expression of 4-HNE was increased (*P* < 0.05) (Figures 7(a), 7(b), 7(c), and 7(d)).

### 3.8. Apoptosis of Cardiac Fibroblasts

**Figure 8:**
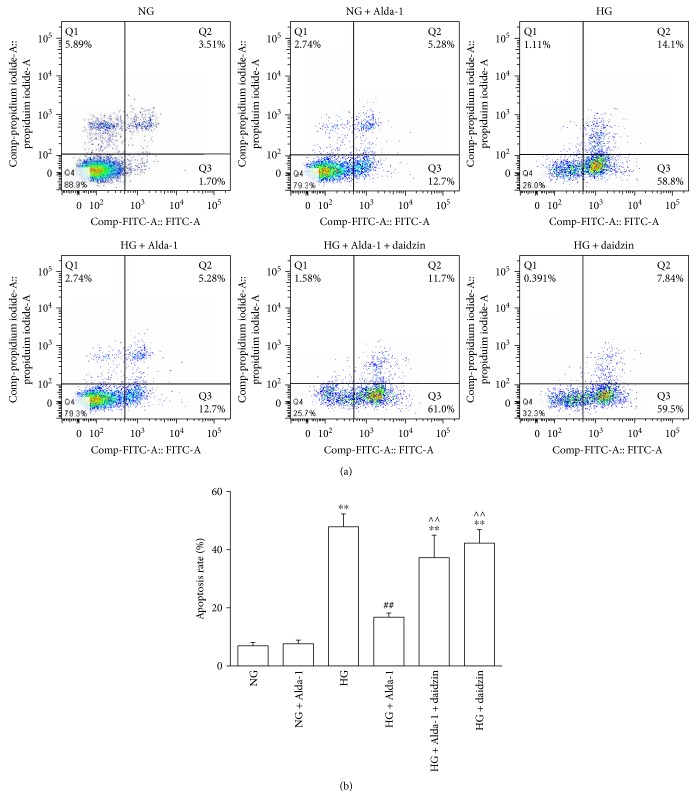
Apoptosis of CFs in different experimental groups. (a) CFs were labeled with Annexin V-FITC and propidium iodide (PI) and analyzed by flow cytometry. Representative pictures were shown. Q1, Q2, Q3, and Q4 represent necrosis, early-stage apoptosis, late-stage apoptosis, and normal cells, respectively. (b) Q2 and Q3 were chosen to analyze the change of apoptosis rate in different experimental groups. Data were presented as mean ± SEM (*N* = 3). ^∗∗^*P* < 0.01 versus NG, ^##^*P* < 0.01 versus HG, ^^^^*P* < 0.01 versus HG + Alda-1.

The CF apoptosis was determined by Annexin V and PI double staining method using flow cytometry. The results indicated that there was no variation of apoptosis rate in NG and NG + Alda-1 group. The apoptosis rate in HG group was increased compared with NG group (*P* < 0.01). The apoptosis rate of in HG + Alda-1 group was decreased compared with HG group (*P* < 0.01). Compared with HG + Alda-1 group, the apoptosis rates in HG + Alda-1 + daidzin and HG + daidzin groups were increased significantly (*P* < 0.01) (Figures 8(a) and 8(b)).

## 4. Discussion

In this study, we characterized the expression and function of ALDH2 on high glucose-induced CF changes. Firstly, double-label immunofluorescence staining, RT-PCR, and Western blot results provided the first evidence that the location of ALDH2 in CFs, which indicated ALDH2, could be necessary in CF function. We observed ALDH2 protein was downregulated accompanied with fibrosis and apoptosis when CFs were cultured with high glucose. Activation of ALDH2 with the specific agonist Alda-1 could restrain the proliferation of CFs cultured with high glucose, reduce the release of ROS and 4-HNE protein expression, decrease oxidative stress overload as well as the expressions of collagen I and collagen III, reverse myocardial fibrosis, and attenuate CFs apoptosis. ALDH2 activity and protein expression were increased at the same time. Taken together, our data indicated that ALDH2 played a protective role in high glucose-induced cardiac fibroblast injury model.

ALDH2 is widely expressed in the heart, brain, liver, kidney, and lung and involved in the occurrence and development of illnesses. Previous studies had shown that ALDH2 attenuated diabetes-induced myocardial injury [17], but is ALDH2 expressed in cardiac fibroblasts? Can ALDH2 protect myocardium against fibrosis? It is rarely reported. We have verified that when treated with a low concentration of ethanol, the nonselective agonist of ALDH2, myocardial fibrosis was improved in diabetic rat [18]. As we know, overproduction of cardiac fibroblasts is one of the important reasons of myocardial fibrosis. In the light of our own and others' research, we speculate that ALDH2 may be expressed in cardiac fibroblasts and is involved in the occurrence of myocardial fibrosis. As we expected, our results showed that ALDH2 was expressed in CFs. It is beneficial for us to investigate the mechanisms of diabetes-induced myocardial fibrosis intensively.

ALDH2 has the protective effects on the various types of cardiovascular injury, such as microvascular injury induced by diabetes, myocardial injury in diabetic rats, and cardiomyocyte injury induced by high glucose [19–22]. Pretreatment with the specific agonist of ALDH2 Alda-1 can improve myocardial ischemia/reperfusion injury in rats by upregulating the expression of ALDH2 [23] and play an important protective role in high glucose-induced myocardial cell injury [13]. Activation of ALDH2 can reduce myocardial fibrosis in diabetic rats and inhibit the expression of JNK (c-Jun N-terminal kinase) which is important in cell proliferation, differentiation, apoptosis, fibrosis, and so on [18]. In this study, we have identified that ALDH2 was expressed in CFs, so we further observe whether activation of ALDH2 also plays a protective effect in high glucose-induced CF injury.

Under normal physiological circumstances, the production and scavenging ability of ROS in intracellular environment are balanced dynamically. Oxidative stress occurred when the production of ROS is far greater than elimination, causing oxidative damage to the DNA and abnormal expression of proteins, finally contributing to cell injury. In recent years, studies showed that the occurrence of myocardial fibrosis in diabetic rats is closely related to oxidative stress. The overproduction of ROS induced by high glucose can attack the polyunsaturated fatty acid (PUFA) of the phospholipid bilayer on the cell membranes, lead to lipid peroxidation, and result in enhanced 4-HNE, an aldehyde product of membrane lipid peroxidation [24]. 4-HNE is one of the representative reactive aldehydes which had been detected in several diseases such as atherosclerosis, diabetes, Parkinson's disease, and cancer [25, 26]. High concentration of 4-HNE can induce cell apoptosis, influence the cell signal transduction, and have a cytotoxic effect [27–29]. ROS has been considered to be a key factor in the development of diabetes and other diseases; the formation of 4-HNE and 4-HNE-protein conjugation had become a marker of oxidative stress in tissues or cells [24, 30–32]. Inhibiting ROS and 4-HNE production can reduce myocardial fibrosis and improve cardiac function in rats with diabetes mellitus [28]. ALDH2 is a key enzyme that metabolizes acetaldehyde and other aldehyde metabolites such as 4-HNE to nontoxic products [33]. In our study, ROS level and 4-HNE protein expression were increased in high-glucose cultured CFs, suggesting high glucose-induced overproduction of ROS and 4-HNE. When given with the specific agonist of ALDH2 Alda-1 in HG-treated CFs, ROS level and 4-HNE protein expression were decreased, while ROS level and 4-HNE protein expression were increased in CFs after treated with the specific ALDH2 antagonist daidzin. These results suggested that ALDH2 played a key role in high glucose-induced oxidative stress, and activation of ALDH2 can eliminate the overproduction of ROS and 4-HNE, then protect CFs against high glucose-induced cell injury.

Fibrosis is an important pathological change in high glucose-induced cardiac fibroblast injury. The main reason of myocardial fibrosis is the accumulation of collagen which disorders the structure of the heart. Collagen I and collagen III are the main collagen components, and among them, collagen I accounts for about 80%, it determines myocardial stiffness, and collagen III accounts for about 12%, which determines myocardial compliance; the balance of collagen I and III plays a significant role in maintaining normal physiological function [34–36]. The oversynthesis of collagen I and collagen III, especially collagen I, may be the main pathological change in myocardial fibrosis. The increased collagen I and collagen III were involved in the myocardial collagen network remodeling, as well as in myocardial fibrosis caused by diabetes mellitus [36]. There was no difference in the expression of ALDH2, collagen I, and collagen III when CFs were treated with Alda-1 compared with control group, it suggested that Alda-1 had no obvious role in ALDH2 expression when CFs were in normal situation. However, when CFs were treated with high glucose, accompanied by the decreases of ALDH2 activity and protein expression, collagen I and collagen III mRNA expressions were increased. It suggested that high glucose can induce the happening of CF fibrosis. When CFs were treated with high glucose and ALDH2 agonist Alda-1, ALDH2 activity and expression were increased while the collagen I and collagen III mRNA expressions were decreased, it suggested that Alda-1 promote the expression of ALDH2, and the increase of ALDH2 can reverse myocardial fibrosis. After treated with daidzin, the ALDH2-specific antagonist, ALDH2 activity and expression were decreased; the synthesis of collagen I and collagen III were increased, aggravating myocardial fibrosis, and further verified the key role of ALDH2. Decreasing ALDH2 expression can induce myocardial fibrosis.

During cardiac fibrosis, complex molecular mechanisms, which play the critical roles in regulating cardiac fibroblast apoptosis, have been shown to be closely related to the occurrence of fibrosis. Apoptotic cells act as the drivers of fibrotic process, it may act directly on CFs, enhancing cellular proliferation and profibrotic phenotypes. High levels of apoptosis are either initiators or perpetuators of the fibrotic response seen in lung fibrosis, liver fibrosis, and chronic myocardium fibrosis accompanied by deposition of extracellular matrix, synthesis of collagen, and fibroblast proliferation [37–39]. High glucose also induced the happening of apoptosis in CFs, in which oxidative stress is the key inducement. The major mechanisms of oxidative stress-induced apoptosis may be as follows [40]: (1) the increased level of ROS leads to NF-*κ*B (nuclear factor kappa-light-chain-enhancer of activated B cells, which was involved in the control of a large number of cellular processes, such as immune and inflammatory responses, cellular growth and development, and apoptosis) activation, which combines with apoptosis-related genes such as c-myc gene to promote transcription and apoptosis; (2) DNA is damaged by ROS, which activates the P53 gene, leading to apoptosis; (3) the increased ROS can directly or indirectly damage the mitochondrial membrane, which leads to the increase of the permeability of the membrane and the activation of the apoptotic protease; (4) ROS activates the MAPK (mitogen-activated protein kinases) signal transduction pathway and then regulates cell proliferation, gene expression, differentiation, mitosis, and cell survival, which eventually activates caspase cascade and induces apoptosis. We have observed that ALDH2 can decrease high glucose-induced overproduction of ROS in a CF model. If ROS is close with apoptosis, can ALDH2 attenuate the occurrence of apoptosis? Our results showed that the apoptosis rate of CFs was increased obviously when cultured with high glucose, while the apoptosis rate was decreased when added Alda-1 in HG group, it suggested that ALDH2 can inhibit the occurrence of apoptosis. When ALDH2 activity was inhibited, the apoptosis rate was increased in HG + Alda-1 + daidzin group, and more verifiably, ALDH2 can regulate the happening of apoptosis. Combined with these data, we speculated that ALDH2 attenuated the damage degree of CF fibrosis through inhibiting apoptosis happening.

In summary, we reported for the first time that ALDH2 is expressed in cardiac fibroblasts. Then, we reported that high glucose can increase oxygen stress reaction, decrease ALDH2 activity and expression, and induce cardiac fibroblast apoptosis and fibrosis. Especially, we reported that the activation of ALDH2 may ameliorate high glucose-induced cardiac fibroblast fibrosis through decreasing oxidative stress and apoptosis. The study may be beneficial to remedy myocardial fibrosis induced by diabetes and other diseases. The intracellular signal transduction mechanisms remain to be further explored.

## Figures and Tables

**Table 1:** Quantitative polymerase chain reaction primers for ALDH2, collagen I, collagen III, and GAPDH.

Gene (accession number)	Sequence	Annealing temperature (°C)	Product (bp)
ALDH2 (NM_032416.1)	Forward 5′-GTG TTC GGA GAC GTC AAA GA-3′ Reverse 5′-GCA GAG CTT GGG ACA GGT AA-3′	62.5	187
Collagen I (NM_053304.1)	Forward 5′-CCA GCG GTG GTT ATG ACT TCA-3′ Reverse 5′-TGC TGG CTC AGG CTC TTG A-3′	59	148
Collagen III (NM_032085.1)	Forward 5′-GGTCACTTTCACTGGTTGACGA-3′ Reverse 5′-TTGAATATCAAACACGCAAGGC-3′	59	201
GAPDH (NM_017008.4)	Forward 5′-ACA GCA ACA GGG TGG AC-3′ Reverse 5′-TTT GAG GGT GCA GCG AAC TT-3′	62	255
